# Pediatric Mesenteric Lipoma: Case Report and Narrative Literature Review

**DOI:** 10.3390/children12040461

**Published:** 2025-04-03

**Authors:** Zeljko Zovko, Alessandro Boscarelli, Daniela Codrich, Rossana Bussani, Francesca Neri, Jürgen Schleef

**Affiliations:** 1 Department of Pediatric Surgery and Urology, Institute for Maternal and Child Health—Istituto di Ricovero e Cura a Carattere Scientifico “Burlo Garofolo”, via dell’Istria, 65/1, 34137 Trieste, Italy; zovko.zeljko@gmail.com (Z.Z.); daniela.codrich@burlo.trieste.it (D.C.); jurgen.schleef@burlo.trieste.it (J.S.); 2 Department of Surgery, University Hospital of Mostar, 88000 Mostar, Bosnia and Herzegovina; 3 Institute of Pathological Anatomy and Histology, Azienda Sanitaria Universitaria Giuliano-Isontina, University of Trieste, 34100 Trieste, Italy; bussani@units.it; 4 Radiology Department, Institute for Maternal and Child Health—IRCCS “Burlo Garofolo”, 34137 Trieste, Italy; francesca.neri@burlo.trieste.it

**Keywords:** mesentery, lipoma, children, mesenteric torsion, management

## Abstract

Introduction: Lipomas are among the most encountered neoplasms in clinical practice, occurring mainly in adults between the fourth and sixth decades of life. Deep-seated lipomas in children are found in the thorax, chest wall, mediastinum, pleura, pelvis, retroperitoneum, and paratesticular area. Herein, we present a case of a three-year-old child with a giant mesenteric lipoma, along with a review of the literature on mesenteric lipomas in childhood. Case presentation: A three-year-old male toddler was referred to our hospital for severe, intermittent abdominal pain. Imaging studies at admission revealed a fat lesion occupying most of the peritoneal cavity and dislocating adjacent structures. An urgent laparotomy was performed. A giant lipoma arising from the mesentery and leading to the torsion of the mesenteric radix was confirmed and completely excised alongside an adherent small tract of jejunum. The child recovered uneventfully and is still being followed-up with no signs of recurrence. Discussion: Lipomas of the mesentery in children are very rare, and they are reported to be more common among children younger than three years of age. Mesenteric lipomas appeared to be more frequent in males than females. Even though they might be asymptomatic, voluminous lipomas can also create a lead point for intermittent torsion of the mass causing ischemia and infarction. Abdominal pain was the most frequent symptom, and the ileum was the tract of bowel more frequently involved by the tumor. Laparotomy was reported to be the preferable approach to safely remove this abdominal mass, especially in case of huge dimensions.

## 1. Introduction

Lipomas are benign tumors of mature fat cells and are typically well-defined, enclosed in a fibrous capsule, and noninvasive. Lipomas are among the most encountered neoplasms in clinical practice. The prevalence is estimated to be around 1% of the entire population, with an incidence rate of around 2.1 per 1000 annually, occurring mainly in adults aged 40 to 60. They can arise anywhere in the body where fat cells are present and may cause variable and nonspecific symptoms, depending on size and location [1,2,3,4].

Lipomas are rare in the first 2 decades of life, may occur as single or multiple tumors, and can be located superficially or deeply in the body. Deep-seated lipomas in children are found in the thorax, chest wall, mediastinum, pleura, pelvis, retroperitoneum, and paratesticular area [5,6]. Particularly, lipomas of the mesentery involving the small bowel are very rare in children [1,6,7,8,9,10,11].

Mesenteric lipomas are slow-growing, soft, mobile masses that do not infiltrate the surrounding tissue; provided they allow bowel passage, they do not cause gastrointestinal symptoms [1,11]. Although mainly asymptomatic, nonspecific symptoms may occur, such as abdominal pain, vomiting, constipation, abdominal distension, and even partial or complete abdominal obstruction or volvulus [1,6,11,12,13].

Herein, we present a case of a 3-year-old male child with a giant mesenteric lipoma. A review of the literature regarding mesenteric lipomas in childhood was also performed.

## 2. Case Presentation

**Figure 1:**
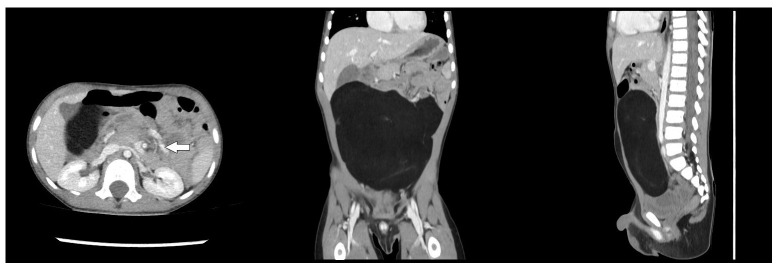
Pre-operative Computed Tomography (TC). Axial (**left**), coronal (**middle**), and sagittal (**right**) scans showing a huge, low-fat-density neoformation and a sign consistent with mesenteric torsion (white arrow).

**Figure 2:**
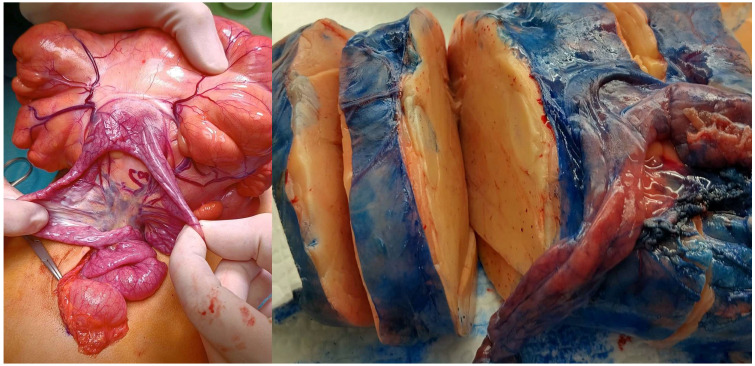
Intraoperative close-up view (**left**) and macroscopic appearance (**right**) of a giant mesenteric lipoma involving a small tract of jejunum.

**Figure 3:**
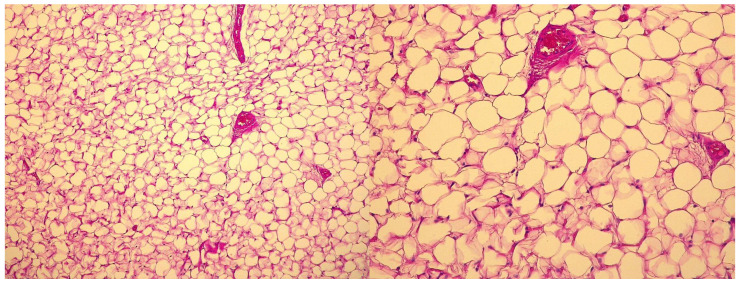
Microscopic images of the mesenteric lipoma taken through a 10X. (**left**) and 20X (**right**) objectives on hematoxylin and eosin-stained histological sections.

A 3-year-old male was referred to our institute for maternal and child health “Burlo Garofolo” of Trieste (Italy) at the beginning of January 2025 with severe, intermittent abdominal pain that started the night before, which raised suspicion of intestinal intussusception. During each painful episode, the child cried inconsolably and then became drowsy or fell asleep. No significant past medical or surgical history was reported, and the family history was unremarkable. The child presented a single episode of vomiting on the day of admission, with no other abdominal symptoms reported in the preceding days. On examination, the child was afebrile with stable vital signs. Abdominal palpation revealed diffuse fullness in the mid-abdomen without well-defined borders. Mild tenderness was noted, but no rebound tenderness or guarding occurred. Routine laboratory tests, including a complete blood count and basic metabolic panel, were within normal limits. Abdominal ultrasound demonstrated a hyperechoic intra-abdominal mass. A subsequent computed tomography (CT) scan confirmed a large, low-fat-density lesion measuring 13.2 × 4.5 × 12 cm, occupying most of the peritoneal cavity and dislocating adjacent structures (Figure 1). Although initial concerns included possible bowel obstruction or intussusception, no conclusive evidence of mechanical obstruction was found. Nonetheless, due to the acute presentation and escalating discomfort, an urgent laparotomy was performed for diagnostic and therapeutic purposes through a median incision under general anesthesia. Intraoperatively, a large, lobulated, yellow, soft mass arising from the mesentery was identified. Notably, a small jejunum tract was tightly adhered to the mass (Figure 2). The tumor was completely excised alongside the adherent small bowel, and an end-to-end anastomosis was created using interrupted absorbable stitches. Further investigation of the abdomen revealed a torsion of the mesentery radix that was recognized as the cause of a slightly bluish coloration of the small bowel loops. Once derotated, the whole small bowel recovered its normal coloration in a few seconds. Preoperative values of tumor markers were within normal ranges, and the pathohistological examination confirmed a giant lipoma 18.5 × 11.4 × 4.2 cm in size, weighing 548 g, not infiltrating the adherent portion of the jejunum (14 cm in length), and with no evidence of malignancy (Figure 3). After the operation, the patient was monitored in our intensive care unit only for the night. The child recovered uneventfully and was discharged home on the 8th postoperative day. At a 7-day follow-up, the patient was in good health with a satisfactory aesthetic result. The child is still being followed up with no signs of recurrence.

## 3. Discussion

Lipomas of the mesentery in children are very rare, although they are among the most common neoplasms of mesenchymal origin. Interestingly, they are reported to be more common among children younger than 3 years [1,2,11,14]. Although they might be asymptomatic, frequent symptoms include progressive abdominal distension, abdominal pain, vomiting, constipation, feeling full after meals, and anorexia. Particularly, lesions exceeding 2 cm can cause abdominal pain, GI bleeding, intussusception, and bowel obstruction [1,11,14]. In 2003, Wolko and colleagues stated that it has not been fully explained how mesenteric lipomas can cause bowel obstruction. Additionally, they suggested that an obstruction may be caused by undue stress on the mesentery from the weight of the mass, which weakens and elongates the mesentery. This can also create a lead point for intermittent torsion of the mass. Furthermore, the twisting may cause tugging of the adjacent bowel, leading to obstruction [15]. However, Rwomurushaka et al. reported a partial intestinal obstruction resulting from extramural compression or a complete intestinal obstruction secondary to small bowel volvulus [2]. Notably, larger lipomas may twist around their vascular pedicle, causing ischemia and infarction [14]. In our case, the patient was transferred to our center in the evening for recurrent episodes of severe abdominal pain started the night before. Remarkably, the child toggled moments of agitation with moments of sudden falling asleep.

Roentgenography may show a well-demarcated globular radiolucent mass clearly outlined by the greater density of the surrounding tissue [5,12]. However, the sonographic appearance of a mesenteric lipoma is that of a well-encapsulated echogenic mass with good through-transmission. Intraperitoneal lipomas can be confidently diagnosed when a homogeneous, highly echogenic encapsulated intra-abdominal mass is detected in children, especially if the mass is radiolucent on conventional radiographs [16]. On CT, the lipoma has typical fat tissue attenuation values from −80 to −120 HU. CT can also determine the omental or mesenteric origin of the lipoma and the precise anatomic features of the tumor. When performed, magnetic resonance imaging usually shows a homogenous signal intensity for the lipoma, identical to fat in all pulse sequences. This can help differentiate the lesion from adjacent structures and depict precise anatomic demarcations [17]. In this case, an abdominal ultrasound evaluation was regularly performed at admission to our emergency department. Based on the results of the US scans and presenting symptoms, an urgent abdominal CT was considered mandatory and it showed features compatible with a giant abdominal fat-containing lesion. Consequently, due to the patient’s symptoms and the dimensions of the lesion, an urgent laparotomy was performed through a midline abdominal incision under general anesthesia. The tumor arising from the mesentery and involving a small tract of the jejunum was effortlessly removed, and an end-to-end anastomosis of the jejunum was fashioned using interrupted absorbable stitches. In addition, the voluminous lesion caused a torsion of the mesentery radix that was recognized and derotated. No abdominal drain was left at the end of the procedure. Post-operative oral feeding was started based on patient’s symptoms and the child was discharged home after full recovery. First follow-up was arranged 7 days after discharge in our surgical department, and then the patient was referred back to the family pediatrician.

To obtain a narrative review of the literature, we searched the PubMed and Google Scholar databases between 1947 and 2024 using the following Medical Subject Heading terms: “lipoma”, “mesentery”, “child”, and “pediatric”. In summary, according to our review of the literature, mesenteric lipomas appeared slightly more frequent in males than females (13 males, 12 females, 1 not reported). The median age at presentation was 48 months (ranging from 9 months to 14 years), as in our case. Abdominal pain was the most frequent symptom, followed by abdominal distension and vomiting. The ileum was the intestinal tract more frequently involved by the tumor. Laparotomy was reported to be the preferable approach to safely remove the abdominal mass [1,2,3,4,5,6,7,8,9,10,11,12,13,14,15,16,17,18,19,20,21,22,23,24,25,26] (Table 1). Notably, Stransky et al. and Ogilvy et al. published articles about mesenteric lipomas in children. However, since these articles could not be fully accessed, we excluded them from our review [27,28]. A few more cases mentioned by Tayeh and colleagues and Turk and colleagues were published in languages other than English; thus, we did not add them to our table [6,11]. Moreover, in their review, Tayeh C et al. mentioned additional papers published in 2012 and 2014, but neither article could be fully accessed; therefore, we did not include them. Additionally, in 2016, Yang et al. published a paper on abdominal masses causing volvulus in children during a 20-year period in their hospital, where they noted five patients who had mesenteric lipomas. Since those cases were not described individually but as a group, we did not include them in this paper [29]. Overall, our findings were in line with the reports of similar cases in the pediatric and adult literature in terms of gender preponderance, age at presentation, prevalent symptoms, imaging techniques, surgical approach, and length of stay (LoS). Conversely, in our case, the intestinal tract involved by the tumor was the jejunum instead of the ileum more frequently reported in the majority of articles [3,30,31].

We acknowledge that our study has a few limitations. The paper is based on those cases accessible in the literature, but true incidence is likely to be higher. Equally we take into account reporting bias due to asymptomatic or sub-acute symptomatic patients.

## 4. Conclusions

Mesenteric lipomas are rare in the pediatric population. They are usually asymptomatic but can cause unspecific symptoms such as abdominal pain, abdominal distension, vomiting, and constipation. Depending on their size and location, they can even lead to intestinal volvulus or obstruction. Although X-rays or ultrasound can provide helpful information, CT usually enables a more accurate description of the mass. Laparotomy with total removal of the lipoma, with or without bowel resection and an end-to-end anastomosis, is the treatment of choice in almost all cases. Particularly, we would warn pediatric surgeons not to underestimate a patient presenting with abdominal pain who alternates moments of agitation with moments of sudden falling asleep to avoid delaying a surgical exploration, with potentially devastating consequences.

## Figures and Tables

**Table 1:** Previous cases of Pediatric Mesenteric Lipoma reported in the literature.

Authors	Year	Age (Months)	Sex	Comorbidities	Symptoms	Symptoms Duration	Imaging	Tumor Size (mm)	Tumor Weight (g)	Localization	Type of Surgery	Intraoperative Findings	Complications	LoS (Days)
Selman J et al. [18]	1947	48	F	none	abdominal mass; nausea; vomiting	4 y	X-ray	NR	NR	ileum	laparotomy	NR	none	NR
Prince T C et al. [8]	1956	10	F	NR	abdominal mass	3 m	X-ray	200	NR	ileum	laparotomy	NR	none	7
Gupta D K et al. [19]	1988	14	F	NR	NR	NR	NR	150	NR	ileum	NR	NR	NR	NR
Prando A et al. [16]	1990	30	M	NR	abdominal mass	NR	X-ray; US	110 × 90 × 50	NR	jejunum	NR	adherent to the wall of small bowel	NR	NR
Kaniklides C et al. [9]	1998	132	M	NR	abdominal pain; constipation	8 y	X-ray; US; CT	220 × 290 × 50	700	mesentery	laparotomy	NR	NR	NR
Ilhan H et al. [5]	1999	36	M	none	abdominal distension	6 m	US; CT	310 × 230 × 120	2050	mesentery	laparotomy	NR	none	6
Wolko J D et al. [15]	2003	108	M	Sorbital Intolerance	abdominal pain	10 d	CT	100 × 150	NR	NR	NR	torsion of the lipoma; small bowel obstruction	none	NR
Ozel S K et al. [12]	2004	84	F	NR	abdominal pain; bilius vomiting	4 d	X-ray; US; CT	180 × 150 × 50	NR	ileum	laparotomy	NR	NR	NR
Cherian A et al. [20]	2004	168	F	NR	abdominal pain; bilius vomiting	8 h	X-ray	160 × 150 × 75	NR	ileum	laporoscopy; laparotomy	volvulus	none	7
Kisra M et al. [21]	2006	168	M	NR	NR	NR	NR	124 × 68 × 110	NR	ileum	NR	volvulus	NR	NR
Srinivasan K G et al. [22]	2009	9	NR	NR	abdominal distension; diarrhoea	3 m	US; CT	NR	1500	omentum; mesentery; transverse colon	laparotomy	NR	NR	NR
Ahmed I et al. [23]	2011	72	M	none	abdominal pain; abdominal distension; constipation	2 y	CT	NR	NR	ileum	laparotomy	NR	none	NR
Turk E et al. [11]	2013	24	F	none	abdominal pain, bilius vomiting	24 h	X-ray; US	160 × 150 × 80	770	ileum	laparotomy	intestinal obstruction with necrosis	none	5
Alireza R et al. [24]	2013	72	F	NR	abdominal pain; nausea; vomiting	2 d	X-ray; US; CT	130 × 30 × 50	NR	ileum	laparotomy	volvulus	none	NR
Tayeh C et al. [6]	2015	24	M	none	failure to thrive; abdominal distension	1 y	X-ray; US	220 × 190 × 90	1620	ileum	laparotomy	NR	none	6
Laguna B A et al. [14]	2015	72	M	Bannayan-Riley-Ruvelbaca Syndrome	abdominal pain	12 h	CT	68 × 42 × 83	NR	mesocolon	laparoscopy	torsion of the lipoma	NR	NR
Hamidi H et al. [17]	2016	72	F	NR	abdominal pain; abdominal distension	4 y	US; CT	280 × 240 × 100	NR	NR	laparotomy	NR	none	NR
Hashizume N et al. [3]	2020	36	F	none	abdominal pain; abdominal distension	NR	US; CT; MRI	80 × 60	NR	ileum	laparotomy	NR	none	NR
Maree G et al. [13]	2020	12	M	NR	abdominal distension; diarrhoea	NR	US; CT	90 × 110	NR	jejunum	laparotomy	NR	none	5
Malik H et al. [7]	2020	72	F	NR	abdominal pain; vomiting; constipation	24 h	X-ray	100 × 80; 20 × 20	NR	ileum	laparotomy	volvulus	none	NR
Azhar M et al. [4]	2021	11	M	none	abdominal distension; constipation; abdominal pain; vomiting	4 m	US; CT	300 × 190 × 120	NR	ileum	laparotomy	small bowel wrapped around tumor	none	5
Hanine D et al. [10]	2021	60	M	NR	abdominal pain	NR	X-ray; US; CT	NR	NR	ileum	laparotomy	NR	none	NR
Mozumder MR et al. [25]	2022	96	F	none	abdominal pain; vomiting	1.5 y	US; CT	85 × 65 × 45	150	ileum	laparotomy	NR	none	NR
Oztas T et al. [1]	2023	48	M	none	abdominal pain; vomiting	NR	X-ray; US; CT	120 × 120 × 70	NR	ileum	NR	volvulus	none	6
Rwomurushaka E S et al. [2]	2024	36	F	NR	abdominal distension; nausea	1 y	US; CT	170 × 170 × 90	NR	small bowel; mesentery	laparotomy	NR	none	3
Garge S et al. [26]	2024	24	M	NR	abdominal distension, abdominal pain, failure to thrive	6 m	X-ray; US; CT	150 × 60 × 100	NR	ileum	laparotomy	NR	none	5

Abbreviations: M = male; F = Female; NR = Not Reported; y = year(S); m = month(S); d = day(S); h = hour(S); US = Ultrasonography; CT = Computed Tomography; LoS = Length of Stay.

## Data Availability

The data that support the findings of this study are available on request from the corresponding author.
